# Development and Validation of a Comprehensive Model to Estimate Early Allograft Failure Among Patients Requiring Early Liver Retransplant

**DOI:** 10.1001/jamasurg.2020.4095

**Published:** 2020-10-28

**Authors:** Alfonso W. Avolio, Antonio Franco, Andrea Schlegel, Quirino Lai, Sonia Meli, Patrizia Burra, Damiano Patrono, Matteo Ravaioli, Domenico Bassi, Fabio Ferla, Duilio Pagano, Paola Violi, Stefania Camagni, Daniele Dondossola, Roberto Montalti, Wasfi Alrawashdeh, Alessandro Vitale, Luciana Teofili, Gabriele Spoletini, Paolo Magistri, Marco Bongini, Massimo Rossi, Vincenzo Mazzaferro, Fabrizio Di Benedetto, John Hammond, Marco Vivarelli, Salvatore Agnes, Michele Colledan, Amedeo Carraro, Matteo Cescon, Luciano De Carlis, Lucio Caccamo, Salvatore Gruttadauria, Paolo Muiesan, Umberto Cillo, Renato Romagnoli, Paolo De Simone

**Affiliations:** 1 Fondazione Policlinico Universitario Agostino Gemelli, IRCCS, Rome, Italy; 2 Università Cattolica del Sacro Cuore, Rome, Italy; 3 Queen Elizabeth Hospital, Birmingham, United Kingdom; 4 Umberto I University Hospital, Rome, Italy; 5 University Hospital, Pisa, Italy; 6 University Hospital, Padua, Italy; 7 Molinette University Hospital, Turin, Italy; 8 S. Orsola-Malpighi University Hospital, Bologna, Italy; 9 Niguarda Hospital, Milan, Italy; 10 ISMETT (Mediterranean Institute for Transplantation and Advanced Specialized Therapies), Palermo, Italy; 11 University Hospital, Verona, Italy; 12 Papa Giovanni XXIII Hospital, Bergamo, Italy; 13 Fondazione IRCCS Ospedale Maggiore Policlinico, Università degli Studi, Milan, Italy; 14 University Hospital, Ancona, Italy; 15 Newcastle Upon Tyne Hospital, Newcastle Upon Tyne, United Kingdom; 16 University Hospital, Modena, Italy; 17 Istituto Nazionale Tumori, IRCCS, and Università degli Studi, Milan, Italy

## Abstract

**Importance:**

Expansion of donor acceptance criteria for liver transplant increased the risk for early allograft failure (EAF), and although EAF prediction is pivotal to optimize transplant outcomes, there is no consensus on specific EAF indicators or timing to evaluate EAF. Recently, the Liver Graft Assessment Following Transplantation (L-GrAFT) algorithm, based on aspartate transaminase, bilirubin, platelet, and international normalized ratio kinetics, was developed from a single-center database gathered from 2002 to 2015.

**Objective:**

To develop and validate a simplified comprehensive model estimating at day 10 after liver transplant the EAF risk at day 90 (the Early Allograft Failure Simplified Estimation [EASE] score) and, secondarily, to identify early those patients with unsustainable EAF risk who are suitable for retransplant.

**Design, Setting, and Participants:**

This multicenter cohort study was designed to develop a score capturing a continuum from normal graft function to nonfunction after transplant. Both parenchymal and vascular factors, which provide an indication to list for retransplant, were included among the EAF determinants. The L-GrAFT kinetic approach was adopted and modified with fewer data entries and novel variables. The population included 1609 patients in Italy for the derivation set and 538 patients in the UK for the validation set; all were patients who underwent transplant in 2016 and 2017.

**Main Outcomes and Measures:**

Early allograft failure was defined as graft failure (codified by retransplant or death) for any reason within 90 days after transplant.

**Results:**

At day 90 after transplant, the incidence of EAF was 110 of 1609 patients (6.8%) in the derivation set and 41 of 538 patients (7.6%) in the external validation set. Median (interquartile range) ages were 57 (51-62) years in the derivation data set and 56 (49-62) years in the validation data set. The EASE score was developed through 17 entries derived from 8 variables, including the Model for End-stage Liver Disease score, blood transfusion, early thrombosis of hepatic vessels, and kinetic parameters of transaminases, platelet count, and bilirubin. Donor parameters (age, donation after cardiac death, and machine perfusion) were not associated with EAF risk. Results were adjusted for transplant center volume. In receiver operating characteristic curve analyses, the EASE score outperformed L-GrAFT, Model for Early Allograft Function, Early Allograft Dysfunction, Eurotransplant Donor Risk Index, donor age × Model for End-stage Liver Disease, and Donor Risk Index scores, estimating day 90 EAF in 87% (95% CI, 83%-91%) of cases in both the derivation data set and the internal validation data set. Patients could be stratified in 5 classes, with those in the highest class exhibiting unsustainable EAF risk.

**Conclusions and Relevance:**

This study found that the developed EASE score reliably estimated EAF risk. Knowledge of contributing factors may help clinicians to mitigate risk factors and guide them through the challenging clinical decision to allocate patients to early liver retransplant. The EASE score may be used in translational research across transplant centers.

## Key Points

**Question:**

Can the individual risk estimation for early allograft failure (EAF) be improved in view of liver retransplant?

**Findings:**

In this multicenter cohort study investigating the association between donor-recipient factors and EAF, a novel Early Allograft Failure Simplified Estimation (EASE) score was developed. The score includes Model for End-stage Liver Disease score, transfused packed red blood cells, and hepatic vessel early thrombosis as well as transaminases, platelet, and bilirubin kinetics as variables on day 10 after transplant. The EASE score outperformed previous model scores, estimating EAF risk with 87% accuracy on day 90 after transplant; EASE was developed on a multicenter Italian database (1609 recipients) and validated on an external UK database (538 recipients).

**Meaning:**

In this study, the EASE score rated the EAF risk (0%-100%) and identified cases at unsustainable risk to be listed for retransplant.

## Teaser

This multicenter cohort study develops and validates a simplified comprehensive model that estimates on day 10 after liver transplant the risk of early allograft failure with 90 days among patients who undergo liver transplant.

## Introduction

Early allograft failure (EAF) is a major determinant of outcome after liver transplant (LT).^1,2,3,4,5,6^ Concerns have been raised on the acceptance of increased-risk donors (eg, elderly, highly comorbid, steatotic, donors after circulatory death [DCD]). The acceptance of individuals with such conditions might offset otherwise achieved survival improvements.^7,8,9,10^ In addition, the introduction of machine perfusion (MP) has led to the acceptance of grafts at higher risk of failure.^11,12,13,14,15^

The wide range of definitions as early allograft dysfunction,^3,5,16,17^ primary dysfunction,^1,18^ initial poor graft function,^1^ posttransplant failure,^6^ primary nonfunction,^1^ and delayed nonfunction^19^ all rely on 2 mutually exclusive alternatives: recovery or failure. The lack of agreement on true indicators and timing for evaluating EAF hampered a shared EAF definition. Recently, 2 studies^17,20^ have highlighted that the recovery after LT is a continuous process punctuated by various events, which may change the prognosis with cascading detrimental effects. However, day 90 has been acknowledged as a reliable time to assess failure-free survival.^6,17,20^

Early allograft failure is the result of a complex interplay between donor- and procurement-related factors in combination with perioperative factors, which all contribute to determine the severity of ischemia-reperfusion injury.^18,21,22^ Early allograft failure may be precipitated by clinical events (eg, graft rejection, drug toxicity, kidney failure, thrombosis of hepatic vessels [THV], or sepsis) that may be negatively associated with patient survival. Timely prediction of EAF is pivotal to identify patients potentially benefiting from a rescue retransplant before severe complications develop and preclude this option.^23,24^ When massive cytolysis and signs of liver failure occur within the first 10 days after LT, the indication for retransplant is evident, independent from evidence of THV. Nevertheless, after this first 10 days, the decision of whether or not to retransplant is frequently challenging.

The indicators of EAF have changed over time from aminotransferase peaks^1,2^ to metabolic factors^3,16,17^ and more recently to a combination of time-dependent kinetic parameters, including aspartate aminotransferase (AST) level, bilirubin level, international normalized ratio, and platelet count. According to this approach, the Liver Graft Assessment Following Transplantation (L-GrAFT) model, a 40-data-entry algorithm based on a 2002 to 2015 cohort from a large-volume North American center, was developed.^20^ However, it has not been validated in external multicenter databases.

Our primary study objective was to develop and validate a simplified but comprehensive model available at day 10 after LT to estimate the risk of EAF. The secondary objective was to identify cases at the highest risk of failure to guide the decision-making process for early retransplant.

## Methods

### Study Design

This is a retrospective multicenter study carried out on prospectively maintained databases identifying adult patients who were submitted to deceased donor LT. First, the L-GrAFT model was validated using an Italian database. Using an L-GrAFT–like method, a novel Early Allograft Failure Simplified Estimation (EASE) score was developed and then internally validated. Afterward, the EASE score was validated using an external UK data set (validation set). A detailed description of EASE model development is provided in the eMethods in the Supplement. This study followed the Strengthening the Reporting of Observational Studies in Epidemiology (STROBE) reporting guideline^25^ and is registered at ClinicalTrials.gov (NCT03858088).^26^ The institutional review board of Policlinico Universitario A. Gemelli IRCCS (coordinating center) approved the study. This board also waived the requirement for obtaining patient consent owing to the retrospective study design.

### Setting

Participants included 14 Italian and 2 UK transplant centers with high (≥70 transplants per year) and intermediate (36-69 transplants per year) activity volume,^27,28^ with a high- to intermediate-volume ratio of 1:1 in derivation and validation sets.

### Population

Both the derivation and validation data sets included consecutive adult patients who had undergone a transplant in 2016 and 2017. Median (interquartile range) ages were 57 (51-62) years in the derivation data set and 56 (49-62) years in the validation data set. Patients with acute liver failure, HIV, or combined, domino, or living donor grafts were excluded.

### Variables and Data Collection

Data collected to develop the EASE score included the following: (1) recipient demographic characteristics (age, sex, and body mass index), primary end-stage liver disease diagnosis, diabetes, wait time, perioperative laboratory results (Model for End-stage Liver Disease [MELD] score at transplant and postoperative day [POD] 1 to 10 AST level, bilirubin level, and platelet count), pretransplant dialysis or mechanical ventilation, packed red blood cell (PRBC) transfusions at LT, and conditions complicating the postoperative course (ie, vascular thrombosis, sepsis, multiorgan failure, other complications, and length of stay); (2) donor demographic characteristics (age and sex); (3) grafts (DCD, donation after brain death [DBD], MP grafts, and macrosteatosis); and (4) surgical procedure characteristics (split, cold ischemia time, warm ischemia time, venovenous bypass, temporary portocaval anastomosis, and intraoperative packing). Data were collected daily in electronic databases by data managers at each center. The area under the curve (AUC) and the rate of change or trend (slope) for AST levels, platelet counts, and bilirubin levels during the first 10 PODs were the calculated variables. For each patient, the AUCs and slopes as well as the AUC square form were recorded.

### Outcome Definition

Early allograft failure was defined as graft failure (need for retransplant or death) for any reason at POD 90.^20^ This definition also captures cases of late-occurring EAF (delayed nonfunction).^19^ We considered as EAF determinants all events potentially leading to EAF, independently if they were or were not strictly associated with ischemia-reperfusion injury. Indeed, vascular (thrombosis of the hepatic artery or portal vein), biliary, toxic, and major hemodynamic events were included because any of them interacting with parenchymal dysfunction can affect graft function recovery and favor graft failure and death. Events potentially leading to EAF were recorded.

The risk of failure was stratified as 5 classes. The highest-risk class was defined as unsustainable, consistent with previous literature,^29^ and the cutoff between classes 4 and 5 was defined as the unsustainable risk cutoff.

Criteria leading to retransplant were based on clinical judgment and included (1) evidence of biochemical signs of a nonfunctioning graft; (2) expected deterioration of other vital functions leading to death; and (3) expected substantial change of prognosis after the second graft.

### Statistical Analysis

To build a comprehensive model, we considered an extensive set of preoperative and intraoperative variables. Owing to the time-dependent incidence of EAF, the potential determinants associated with graft failure were investigated at different time intervals (PODs, 2-15, 2-30, 2-60, and 2-90) using univariate Cox regression analysis according to the L-GrAFT method.^20^ Logistic regression analysis was then used. The backward stepwise procedure for variable selection was initially adopted. Variables were then tested using a nonautomatic approach. The discrimination ability of the final model was investigated using the receiver operating characteristic (ROC) curve method. The discrimination ability was also confirmed through internal validation using bootstrap resampling and through external validation using the UK data set. Two hundred bootstrapped samples were taken with replacement from the original data set, with each bootstrapped sample containing 1000 cases. The goodness of fit of the new score was assessed using the Hosmer-Lemeshow test. The calibration of the final model in the derivation and external validation sets was evaluated using the calibration-BELT method. Details are reported in eFigure 4 in the Supplement.

We initially tested β coefficients of the L-GrAFT model derived from 40 data entries, validating the model. Then, we reduced the number of entries by recording only data at specified PODs.

Four subsequent logistic models (1-4) were developed in the derivation set to reduce the number of data entries, improving the Harrell C statistic and including additional factors. Five additional models (5-9) were tested to investigate the impact of THV and of DCD and MP grafts. Models 5 to 9 were adjusted for transplant center volume. Models 1 to 4 were internally validated by bootstrap, and models 5 to 9 were tested in the external validation set. The final simplified comprehensive model (model 9) was selected for a low number of data entries (17) and the highest C statistics in both data sets. All modeling, data, and statistical analyses were conducted using SPSS, version 25.0 (IBM SPSS) and Stata, version 14.0 (StataCorp).

## Results

**Figure 1.**
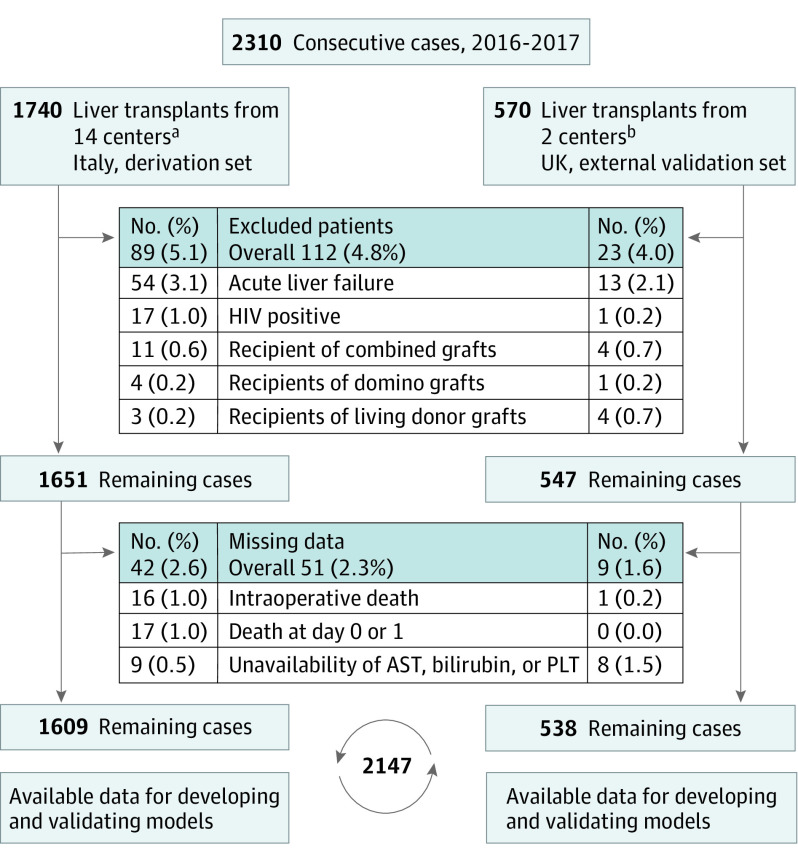
Patient Flow Diagram Patients accrued in the derivation and external validation sets are displayed separately. AST represents aspartate aminotransferase; PLT, platelet count. ^a^Included were 7 high-volume transplant centers and 7 intermediate-volume centers. During the 11- to 90-day period, 1 patient in the derivation set was lost to follow-up. During the 91- to 730-day period (24 months), 5 patients were lost to follow-up. ^b^Included were 1 high-volume transplant center and 1 intermediate volume center.

The initial assessment included 1740 consecutive patients from the Italian database, and 131 patients were excluded (Figure 1). Thus, the derivation set included 1609 patients from this set (Table 1). The external validation set included 538 patients who underwent transplants at 2 UK centers. Figure 1 illustrates patient flow in both data sets. The characteristics of each data set are shown in eTable 1 in the Supplement. On day 90, the incidence of EAF was 110 of 1609 patients (6.8%) in the derivation set and 41 of 538 patients (7.6%) in the external validation set.

**Table 1.** Study Population Characteristics in the Derivation Data Set After Exclusion of Patients With Missing Data for the Parameters Used in the Models^a^

Characteristic	No. (%) of patients	Median (IQR)	Total No. of patients included	No. (%) of patients with missing data
Donor data				
Age, y				
Mean (SD)	61.9 (17.5)	65 (51.2-76.0)	1606	3 (0.2)
≥85	64 (4.0)	NA	1606	3 (0.2)
Male sex (donor)	920 (57.2)	NA	1609	0
Split	50 (3.1)	NA	1609	0
DCD^b^	26 (1.6)	NA	1609	0
MP grafts^b^	80 (5.0)	NA	1609	0
Cold perfusion	60 (3.7)	NA	1609	0
Normothermic perfusion	20 (1.2)	NA	1609	0
Macrosteatosis (30% and higher)	38 (3.5)	NA	1085	524 (32.6)
MP grafts	7 (12.1)	NA	58	
Non-MP grafts	31 (3.0)	NA	1027	
Recipient data				
Age, mean (SD), y	55.5 (9.2)	57 (51-62)	1609	0
Male sex	345 (21.4)	NA	1609	0
BMI				
Mean (SD)	25.7 (4.0)	25.3 (23.0-28.1)	1596	13 (0.8)
<18.5	46 (2.9)	NA	1596	13 (0.8)
>30	234 (14.7)	NA	1596	13 (0.8)
Main indication		NA	1607	2 (0.1)
HCV	643 (40.0)	NA		
HBV	230 (14.3)	NA		
Autoimmune hepatitis	31 (1.9)	NA		
Colestatic diseases	99 (6.2)	NA		
Alcoholic cirrhosis	355 (22.1)	NA		
Other indication	249 (15.5)	NA		
HCC (T2-T3) coindication	715 (44.4)	NA	1609	0
Waiting time in HCC T2-T3, mo	5.5 (10.5)	1.9 (0.6-5.8)	715	0
MELD, mean (SD)				
All patients	15.8 (8.3)	14.0 (9.0-19.9)	1609	0
HCC T2-T3	12.5 (6.4)	10 (8-15)	715	0
Cirrhosis and HCC T1	18.4 (8.7)	16 (12-23)	894	0
Grade 3-4 portal thrombosis (Yerdel)	26 (1.6)	NA	1609	0
Preoperative kidney support	24 (1.5)	NA	1609	0
Preoperative lung support	12 (0.7)	NA	1609	0
Packing for damage control	31 (1.9)	NA	1609	0
VVBP	358 (22.2)	NA	1609	0
Temporary portocaval anastomosis	42 (2.6)	NA	1609	0
CIT, min	420.1 (108.0)	418.5 (357.6-480.4)	1555	54 (3.4)
WIT, min	45.3 (22.1)	42.5 (26.0-60.0)	1327	282 (17.5)
Match and outcome data				
D-MELD	965.5 (560.4)	825.5 (561.4-1236.6)	1606	3 (0.2)
Clavien-Dindo 3b or higher	330 (20.5)	NA	1609	0
Length of stay, d				
Hospital	22.6 (26.9)	26.0 (15.0-60.0)	1574	35 (2.2)
ICU	5.9 (9.9)	10.0 (3.0-24.0)	1555	54 (3.4)

Abbreviations: BMI, body mass index (calculated as weight in kilograms divided by height in meters squared); CIT, cold ischemia time; DCD, donation after cardiac death; D-MELD, donor age × MELD; HBV, hepatitis B virus; HCC, hepatocellular carcinoma; HCV, hepatitis C virus; ICU, intensive care unit; IQR, interquartile range; MELD, Model for End-stage Liver Disease; MP, perfusion machine; NA, not applicable; VVBP, venovenous bypass; WIT, warm ischemia time.

^a^ Missing data and their percentages are also reported.

^b^ Machine perfusion was used in 23 of 26 DCD grafts (88.5%) and in 57 of 1583 donations (3.6%) after brain death grafts.

### EASE Score and Probability Function

**Figure 2.**
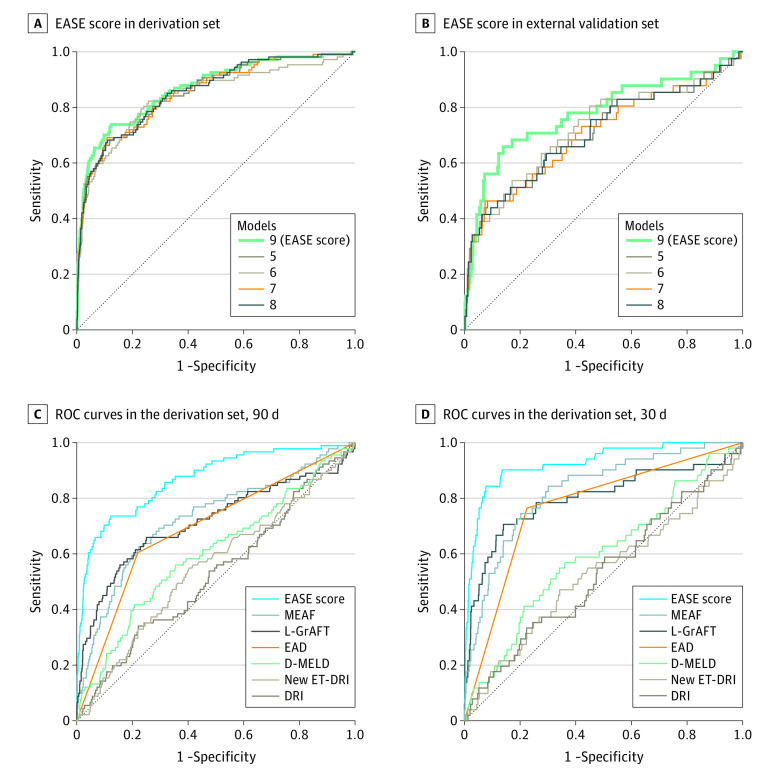
Receiver Operating Characteristic (ROC) Curves A, B. The ROC curves for the Early Allograft Failure Simplified Estimation (EASE) score (final model 9) and other models (5, 6, 7, and 8) at 90 days in the derivation set and in the external validation set. C, The ROC curves in the derivation set. D, The ROC curves for the EASE score developed at 90 days and applied at 30 days and for other estimated scores in the derivation. D-MELD indicates donor age × Model for End-stage Liver Disease; DRI, Donor Risk Index; EAD, Early Allograft Dysfunction; L-GrAFT, Liver Graft Assessment Following Transplantation; MEAF, Model for Early Allograft Function; and New ET-DRI, New Eurotransplant Donor Risk Index.

**Figure 3.**
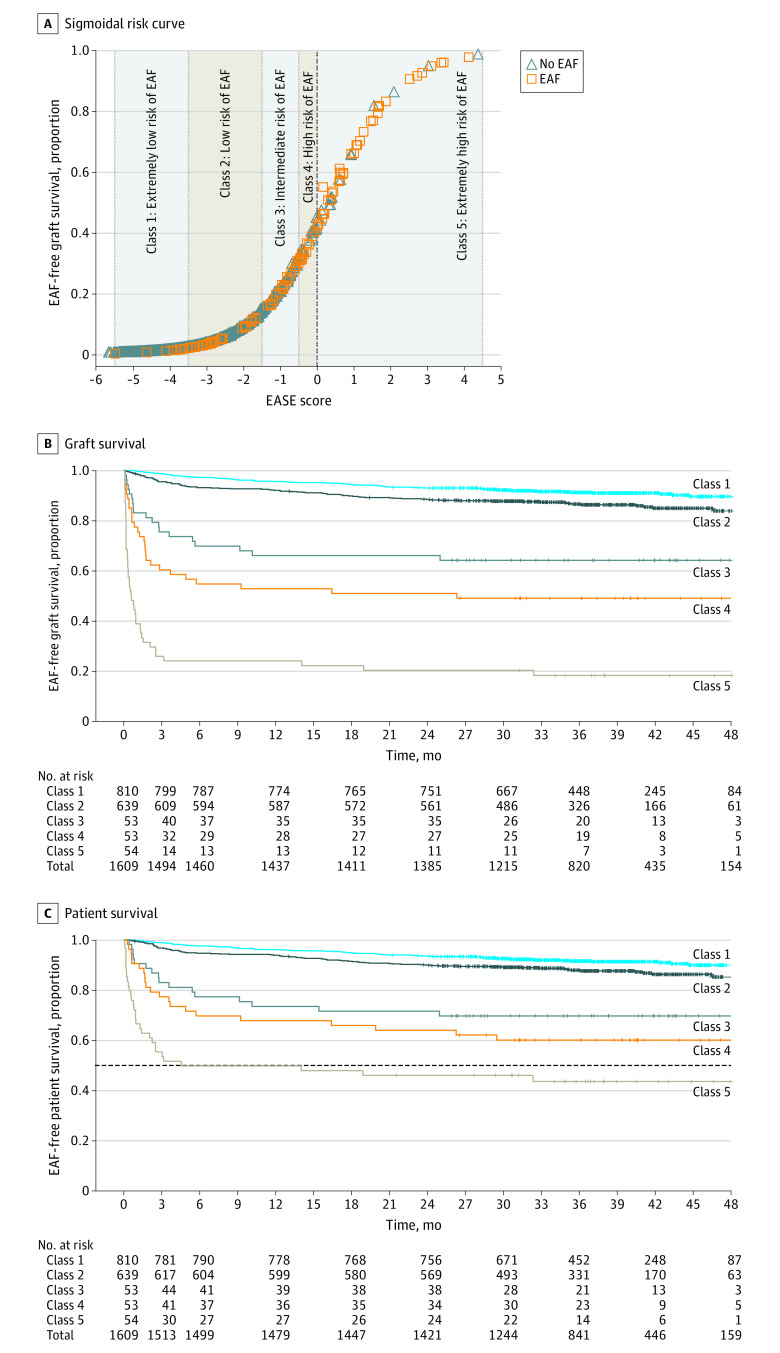
Early Allograft Failure Simplified Estimation (EASE) Score and Kaplan-Meier Survival Curves A, Sigmoidal day 90 early allograft failure (EAF) distribution according to the EASE score in 1609 evaluated patients. Five different risk classes are identified, with the dashed central line denoting the threshold for an unsustainable EAF risk. The constant obtained by logistic regression analysis was increased by 0.3060 to calibrate the unsustainable risk cutoff at the 0 threshold. B and C, Early allograft failure–free graft survival and patient survival according to the 5 EASE score risk classes are shown. The dashed line between classes 4 and 5 in panel C representing the extremely high-risk threshold (unsustainable risk cutoff) indicates the poor survival of patients in extremely high-risk class 5. The extremely high-risk class and the unsustainable risk cutoff indicate the threshold that mandates prompt retransplant.

After preliminary Cox analysis (eFigure 1 in the Supplement), several logistic models were developed. The final model estimation of EAF at 90 days (model 9, EASE score) was identified (Figure 2A and B; eTable 2 and eTable 3 in the Supplement). The EASE score was calculated using 17 data entries: AST on PODs 1, 2, 3, 7, and 10; platelet counts on PODs 1, 3, 7, and 10; bilirubin levels on PODs 1, 3, 7, and 10; MELD score; PRBC transfusions at LT; THV; and transplant center volume. The probability function was obtained by plotting the probabilities of EAF at 90 days against the results of the logistic risk function (Figure 3A). Five EAF risk classes were defined according to the percentile distribution as in the original L-GrAFT study^20^: class 1, extremely low risk (0-49.9 percentile); class 2, low risk (50.0-89.9 percentile); class 3, moderate risk (90.0-93.2 percentile); class 4, high risk (93.3-96.5 percentile); and class 5, extremely high risk (96.6-99.9). The line between classes 4 and 5 represents the extremely high-risk threshold (unsustainable risk cutoff). The risk curve at day 90 and the unsustainable risk cutoff are reported (Figure 3A).

The EASE score was derived as follows: EASE score = 0.958 + (0.044 × MELD score at transplant) + (0.065 × PRBC) + (2.567 × thrombosis on days 1-10) + [0.000534 × AUC^2^ for ln (AST level) on days 1, 2, 3, 7, and 10] + [−0.093 × AUC for ln (platelet count) on days 1, 3, 7, and 10] + [−7.735 × slope for ln (platelet count) on days 1, 3, 7, and 10] + (0.735 × slope for bilirubin level on days 1, 3, 7, and 10) + (−0.402 × high-volume center). The Hosmer-Lemeshow goodness-of-fit statistic was 0.883.

Five representative cases from the study population are described in eTable 4 in the Supplement. The EAF risk ranged from 26% to 96% and depended on interactions among risk factors, which could be detrimental or protective.

### Kaplan-Meier Curves

The graft survival and patient survival curves are reported in Figure 3B and C. The differences across the 5 EAF survival curves at 90 days were significant. Even though the EASE score was designed to identify the EAF risk at 90 days, its performance up to 48 months is also illustrated. After 24 months from transplant, the spread between patient and graft curves increased for EASE class 4 and increased more for EASE class 5. In class 5, the 48-month survival rate was 43.9% (95% CI, 30.2%-57.7%) for patients and 18.3% (95% CI, 6.1%-28.1%) for grafts. Overall survival rates among patients were 95.2% (95% CI, 94.1%-96.2%) at 3 months, 88.7% (95% CI, 87.1%-89.9%) at 24 months, and 84.6% (95% CI, 82.2%-87.1%) at 48 months. Graft hazard curves and patient hazard curves are shown in eFigure 2 in the Supplement. The median (interquartile range) follow-up was 36.4 (30.6-42.7) months.

### Internal and External Validation

Comparisons of EASE with L-GrAFT,^20^ Model for Early Allograft Function Scoring (MEAF),^17^ Early Allograft Dysfunction,^16^ donor age × recipient biochemical Model for End-stage Liver Disease (D-MELD),^29^ New Eurotransplant Donor Risk Index (ET-DRI),^30^ and DRI^6^ are given in Table 2. In the ROC curve analysis, the EASE score outperformed all of the aforementioned scores without 95% CI overlap (C statistic, 0.87; 95% CI, 0.83-0.91; Figure 2C). The C statistic was internally validated through bootstrapping (eTable 5 in the Supplement). We also tested the day 90 EASE model specifically in patients with or without hepatocellular carcinoma. The C statistics were 0.88 (95% CI, 0.77-1.00) for patients with hepatocellular carcinoma and 0.88 (95% CI, 0.73-0.94) for patients without hepatocellular carcinoma.

**Table 2.** Characteristics of EASE Score and Other Published Scores^a^

Characteristic	DRI,^6^ 2006	EAD,^16^ 2010	D-MELD,^29^ 2011	New ET-DRI,^30^ 2012	MEAF,^17^ 2015	L-GrAFT,^20^ 2018	EASE 2020
Object of score	Donor quality	Graft quality	Donor-recipient match	Donor quality	Graft recovery	Graft recovery	Graft recovery
End point	Graft failure	Graft dysfunction	Graft failure/patient death	Graft failure	Graft failure	Graft failure	Comprehensive graft failure
Cutoff	≥2	4	>1628/>1628	>2	≥8	>1.3	>0
% Of estimated cases	80% at 90 d	75% at 180 d	84%/86% at 90 d	79% at 90 d	70% at 90 d	16% at 90 d	28% at 90 d
Day of evaluation in relation to LT	Intraoperative	7	−1	Intraoperative	3	10	10
Donor							
Age	X		X	X			
g-GT				X			
Race (White vs African American)	X						
Height	X						
Cause of death (vascular vs other)	X			X			
DCD	X			X			
Partial or split	X			X			
Recipient							
MELD score at transplant			X				X
Transplant							
Location (local, regional, or national)	X			X			
Cold ischemia time	X			X			
Rescue allocation				X			
Packed red blood cells							X
High-volume center (vs intermediate-volume)							X
After transplant							
INR >1.6 at day 7		X					
Bilirubin >10 mg/dL at day 7		X					
ALT or AST >2000 U/L at day 7		X					
ALT maximum from day 1 to day 3					XXX		
INR maximum from day 1 to day 3					XXX		
Score bilirubin on day 3					XXX		
AST from day 1 to day 10						XXXXXXXXXX	
Bilirubin, daily from day 1 to day 10						XXXXXXXXXX	
Platelets, daily from day 1 to day 10						XXXXXXXXXX	
INR maximum from day 1 to day 10						XXXXXXXXXX	
AST on days 1, 2, 3, 7, and 10							XXXXX
Platelets on days 1, 3, 7, and 10							XXXX
Bilirubin on days 1, 3, 7, and 10							XXXX
Vascular thrombosis within days 1-10							X
No. of variables	8	3	2	8	3	4	7^b^
Total No. of determinations	8	3	2	8	9	40	17
Discrimination ability at 90 d							
In the derivation set	Not reported	0.72^c^	0.70 and 0.64	0.63	Not reported	0.85	0.87
In the validation set or external data set^d^	0.57^d^	0.63^31^^,^^c^^,^^d^	0.72 and 0.64	0.58^31^^,^^d^	0.73^32^^,^^d^	0.71^e^	0.78

Abbreviations: ALT, alanine aminotransferase; AST, aspartate aminotransferase; DCD, donation after cardiac death; D-MELD, donor age × Model for End-stage Liver Disease; DRI, Donor Risk Index; EAD, Early Allograft Dysfunction; EASE, Early Allograft Failure Simplified Estimation; g-GT, gamma-glutamyl transpeptidase; L-GrAFT, Liver Graft Assessment Following Transplantation; INR, international normalized ratio; LT, liver transplant; MEAF, Model for Early Allograft Function; MELD, Model for End-stage Liver Disease; New ET-DRI, New Eurotransplant Donor Risk Index.

SI conversion factors: To convert bilirubin to μmol/L, multiply by 17.104; ALT and AST to μkat/L, by 0.0167

^a^ Scores are different in terms of object, end point, cutoff, number of factors used and total number of entries as well as the discrimination ability at 90 days. The L-GraFT and EASE scores presented the highest C statistic (0.85 and 0.87, respectively). However, the number of data entries necessary to calculate is 17 for EASE and 40 for L-GrAFT. X indicates the characteristics included in the score, multiple X’s indicate data collected at multiple days, and number of X’s indicates the number of days.

^b^ The number of variables is 7; however, the number of factors included in the model is 8 because the platelet count is included as the area under the curve and as slope.

^c^ At 180 days.

^d^ External data set.

^e^ In Figure 2C and eTable 5 of the Supplement.

The EASE score was then validated on the external UK database (C statistic, 0.78; 95% CI, 0.69-0.87) (Figure 2B). The performance of the EASE score in the derivation set was similar to that of models not including THV among the covariates or excluding patients with THV, DCD, or MP in various combinations. However, in the external validation set, the EASE score including these variables outperformed models 5 to 8.

### Performance of EASE Score at Earlier Times

The C statistic of the EASE score was also calculated for PODs 15, 30, and 60. The EASE scores achieved even higher performances than for POD 90 (Figure 2D; eFigure 3 in the Supplement).

### Number of Early Graft Losses and Estimate of Additional Graft Requirements in Patients With Extremely High Risk

Among 54 grafts in patients at extremely high risk for EAF, 23 grafts were lost during the initial 10 PODs (6 deaths and 17 liver retransplants). Of the remaining 31 grafts, 17 were lost during the 11th to 90th POD, and only 14 survived 90 or more days. Altogether, estimating that 50% to 75% of 18 grafts with dismal prognosis on day 11 could be considered suitable for retransplant, the additional requirement would be 8.5 to 12.8 grafts in 2 years (ie, 0.5%-0.8% of total grafts).

## Discussion

To our knowledge, this is the first large, multicenter, validated study to envisage the outcome of patients with EAF in view of liver retransplant. The EASE score has been designed to estimate EAF at 90 days and results from a logistic model that includes 8 covariates. Four are kinetic variables obtained during the first 10 days after LT. The remaining are MELD scores at transplant, intraoperative PRBC transfusion requirement at LT, occurrence of THV within 10 days, and transplant center volume. The EASE score improves the day 90 estimation ability of other models, including Early Allograft Dysfunction, MEAF, and L-GrAFT.^16,17,20^

The availability of risky grafts prompts the transplant community to pursue a new comprehensive definition of EAF. Following initial EAF definitions,^1,2^ the discrimination ability of EAF estimation improved through multivariate models,^3,5^ leading to a binary early allograft dysfunction definition.^16^ Modern approaches exploit the dynamic changes in a few posttransplant recipient parameters, resulting in a substantial improvement in comparison with previous algorithms.^17,20^ In particular, L-GrAFT captures 3 main aspects of graft damage and recovery: cytolysis (in line with the historical definitions of EAF), inability to sufficiently clear bilirubin, and endothelial activation with platelet consumption.^33,34^ Adopting a similar approach, the EASE score simplifies and refines the L-GrAFT, including additional variables expressing pretransplant severity of liver disease (MELD), surgical complexity (PRBC), and THV. On the basis of the literature, we used AST level instead of alanine aminotransferase level,^4,13,20,35,36^ whereas the international normalized ratio was not significant and therefore not entered into the final model. We selected the EASE score among the 9 developed models for the high C statistic in the derivation and validation sets. This model includes all graft categories, encompassing different possible scenarios.

The EASE score addresses the unmet clinical need of identifying patients at the 10th POD for whom an early retransplant is the sole option. Previous studies and clinical experience suggest better outcomes for liver retransplant when performed earlier.^23,24,37,38,39^ Listing a patient for retransplant is challenging.^23,24,40,41^ The decision depends on several issues, which may change day by day. Indeed, a reluctance to retransplant may exist in the absence of clear and immediate signs of graft failure, delaying for several weeks the decision to relist. Likewise, postponing retransplant leads to an increasing number of patients who are finally unfit for retransplant due to the development of medical complications (eg, kidney failure, respiratory complications, sepsis, and multiple organ failure) that preclude successful retransplant and definitely lead to death. Moreover, the increased availability of DCD, steatotic, and MP organs makes crucial the reliable prediction of the EAF risk.^11,13,21^

The EASE score clearly identifies patients on POD 10 with a very high EAF risk, which is, indeed, unsustainable. The availability of a robust tool to timely estimate EAF is therefore highly desirable to prevent clinicians from missing the window of opportunity for retransplant among their patients.

Including MELD, PRBC, and THV among the EAF determinants undoubtedly represents a major difference from all previous algorithms. Bilirubin level and international normalized ratio in the pre-MELD era and, more recently, MELD itself have been associated with donor quality and have been identified as cofactors of primary dysfunction or EAF.^3,5,29,42,43,44^ The MELD score estimates EAF in univariate analysis and, with a lower odds ratio due to other competing factors, in multivariate analysis. The result is more evident in patients without hepatocellular carcinoma with high MELD scores and severe decompensation of liver function. The inclusion of PRBC is innovative, too. We translated a well-known concept from general surgery to transplant surgery: high PRBC consumption reflects increased risk and poorer outcomes.^45^

In the derivation set, models not including THV among the covariates displayed negligible differences compared with the EASE score. However, the EASE score outfit all other models in the validation set. There are several reasons for considering THV as a potential EAF determinant. First, in the included patients, the majority of thromboses occurred during the first 72 hours, and it is difficult to decipher to what extent cytolysis depended on ischemia-reperfusion injury or to THV-associated ischemic damage. Ischemia-reperfusion injury may act as a cofactor of concurrent vascular problems, and the impact of arterial thrombosis may range from minimal to severe, whereas isolated portal thrombosis is generally better tolerated. Evidence from other studies reports that up to 51% of cases of hepatic artery thrombosis respond to prompt revascularization, fibrinolysis, or anticoagulation therapy without resulting in graft failure.^46,47^ Moreover, our study population included patients without THV on day 10 who later developed thrombosis as well as patients with THV who responded to revascularization and to fibrinolysis/anticoagulation therapy. Conversely, moderate parenchymal dysfunction may be worsened by additional thrombotic events. In univariate analysis, either arterial or portal thrombosis significantly estimated EAF, with a higher odds ratio for arterial than portal thrombosis. Nevertheless, in multivariate analysis, a significant risk for EAF was detected only when arterial and portal thrombosis cases were combined. Notably, we failed to show a predictive role for donor age or for DCD or MP grafts. Although all these factors might lead to graft loss in a minority of cases,^13,14^ higher graft failure rate has been reported later than 90 days owing to ischemic cholangiopathy. We also failed to show that donor macrovesicular steatosis may be associated with detrimental effects. This failure may be due to the presence of competing recipient variables, such as AST or bilirubin levels.

Finally, liver retransplant remains the only available treatment of EAF due to either parenchymal or vascular causes. Different from previous scores, the EASE score includes almost all components of the process leading to graft failure, and we believe that this characteristic might explain a better performance of the EASE score compared with less multifaceted scores. We also tested the EASE score on PODs 15, 30, and 60, obtaining better C statistic results compared with 90 days. We suppose that the estimation ability of EASE slightly decreases with the increase of the incidence and severity of infections. Septic events are not considered by this score. Even though preoperative sepsis, high lactate levels, and high sequential organ failure assessment scores at intensive care unit admission are well-known predictors of graft failure and futility,^48,49^ in the posttransplant period, sepsis generally occurs after POD 10, when EASE is calculated. However, the excellent C statistic on POD 15 supports the prompt referral to retransplant of few controversial high-risk cases, with negligible harm to the waiting list.^50^

The EASE score enables the rating of the EAF risk in a range from 0% to 100%. It can be helpful to evaluate the efficacy of standard, DCD, or MP grafts in critical appraisal of single-center or multicenter analyses. The information provided by the EASE score, particularly in the 2 upper EAF risk classes, might also be useful to weigh the retransplant risks against contraindications for relisting. The EASE model performs best as a continuous score. However, for practical reasons, we identified the boundary between class 4 and class 5 as the unsustainable threshold for EAF risk (EASE score >0).

In addition, the EASE score enables the stratification of patient survival up to 4 years. The 48-month patient survival rate of EASE class 5 is 44%, and we could hypothesize an expected 5-year survival rate slightly higher than 40%. In other words, although late retransplant can rescue some grafts lost for sequalae of ischemic cholangiopathy, patient survival still remains below the futility threshold of 50% at 5 years.^25,29,51,52^ Considering that late retransplant is a demanding and challenging procedure, the EASE risk calculation on day 10 may allow for an early and efficacious retransplant indication, reducing the need for late retransplant due to ischemic cholangiopathy.

The main strength of EASE is the great estimation of failure at 90 days, already calculated shortly after the first week of transplant, which is better than any other model. Secondary strengths are the large number of transplants, the solid statistical method, and the recent observational period (2016-2017). Moreover, the EASE score was developed using a multicenter design and was subjected to 2 types of validation (internal, compared with other models by ROC bootstrapping; and external, using data from 2 UK centers). Furthermore, the present study introduces the innovative concept of the unsustainability of extremely high-risk situations, suggesting for them listing for prompt retransplant. Although it evolved from L-GrAFT, EASE requires fewer data entries and appears more user friendly in clinical practice.

### Limitations

Our study is not free from limitations. First, the data sets include retrospective series of liver transplants across Italy and the UK, and the proposed algorithm cannot straightaway be extended to countries with potential differences in donor and recipient characteristics. Second, owing to the exclusion of some recipient categories (ie, acute liver failure; patients with HIV; patients with combined grafts; and both domino and living graft recipients), the EASE score might not apply to these recipients. Third, the EASE score is not useful to guide the indication to retransplant during the first 10 days because it is calculated on POD 10. For the first 10 days, additional tools are needed. Moreover, the number of PRBC units transfused and the management of vascular thrombosis may depend on transplant center policies and anesthesiology regimens.^53,54^ Finally, our study was not extended to low-volume centers, and the application of the EASE score in this setting needs to be verified.

## Conclusions

Refining the L-GrAFT algorithm, we increased the ability to estimate EAF through an EASE score, a new simplified comprehensive model. The EASE score will support transplant surgeons and hepatologists in the decision-making process of listing patients for retransplant. EASE represents a valuable tool to quantify early graft function, and the highest-risk class may serve as an end point in future trials. Further national and international studies are warranted.

## Notes

Supplement. **eTable 1.** Donor’s and Recipient’s Data in the Derivation and Validation Sets **eTable 2.** Univariate Analysis and Multivariate Analysis of Factors Predictive of EAF at 90 Days **eTable 3.** C-Statistics of EASE Score (Final Model 9) and Other Models (5, 6, 7, 8) at 90 Days in the Derivation Set and in the External Validation Set **eTable 4.** Representative Cases With Relative EASE Scores and Allograft Outcomes **eTable 5.** C-statistics of EASE Score (Which Is Calculated at 90 Days) and Other Prognostic Scores in the Derivation Set, EASE Score Shows the Highest C-Statistic at 90 Days **eFigure 1.** Changes in Cox-Estimated Hazard Ratio (HR) of Significant Covariates (AUC^2^ in PLT, Slope in PLT, Slope in Bilirubin, MELD, PRBC, Early Thrombosis of Hepatic Vessel) **eFigure 2.** Kaplan-Meier EAF-Free Graft Hazard (A) and Patient Hazard (B) According to the 5 EASE Score Risk Classes **eFigure 3.** Trend of C-Statistic During the Post-Operative Period **eFigure 4.** Calibration BELT Tests **eMethods.** Detailed Description of Statistical Analysis and Workflow to Develop the Final Model **eReferences.**
