# Supplementary material for: Development and Validation of a Comprehensive Model to Estimate Early Allograft Failure Among Patients Requiring Early Liver Retransplant
Source: JAMA Surg. 2020 Oct 28;155(12):e204095. doi: 10.1001/jamasurg.2020.4095 (PMC7593884; doi:10.1001/jamasurg.2020.4095)
Supplement: Supplement. — eTable 1. Donor’s and Recipient’s Data in the Derivation and Validation Sets eTable 2. Univariate Analysis and Multivariate Analysis of Factors Predictive of EAF at 90 Days eTable 3. C-Statistics of EASE Score (Final Model 9) and Other Models (5, 6, 7, 8) at 90 Days in the Derivation Set and in the External Validation Set eTable 4. Representative Cases With Relative EASE Scores and Allograft Outcomes eTable 5. C-statistics of EASE Score (Which Is Calculated at 90 Days) and Other Prognostic Scores in the Derivation Set, EASE Score Shows the Highest C-Statistic at 90 Days eFigure 1. Changes in Cox-Estimated Hazard Ratio (HR) of Significant Covariates (AUC2 in PLT, Slope in PLT, Slope in Bilirubin, MELD, PRBC, Early Thrombosis of Hepatic Vessel) eFigure 2. Kaplan-Meier EAF-Free Graft Hazard (A) and Patient Hazard (B) According to the 5 EASE Score Risk Classes eFigure 3. Trend of C-Statistic During the Post-Operative Period eFigure 4. Calibration BELT Tests eMethods. Detailed Description of Statistical Analysis and Workflow to Develop the Final Model eReferences. [file jamasurg-e204095-s001.pdf]

Supplementary Online Content

Avolio AW, Franco A, Schlegel A, et al. Development and validation of a comprehensive model to estimate early allograft failure among patients requiring early liver retransplant. *JAMA Surg*. Published online October 28, 2020. doi:10.1001/jamasurg.2020.4095

eTable 1. Donor's and Recipient's Data in the Derivation and Validation Sets

eTable 2. Univariate Analysis and Multivariate Analysis of Factors Predictive of EAF at 90 Days

eTable 3. C-Statistics of EASE Score (Final Model 9) and Other Models (5, 6, 7, 8) at 90 Days in the Derivation Set and in the External Validation Set

eTable 4. Representative Cases With Relative EASE Scores and Allograft Outcomes

eTable 5. C-statistics of EASE Score (Which Is Calculated at 90 Days) and Other Prognostic Scores in the Derivation Set, EASE Score Shows the Highest C-Statistic at 90 Days

eFigure 1. Changes in Cox-Estimated Hazard Ratio (HR) of Significant Covariates (AUC^2 in PLT, Slope in PLT, Slope in Bilirubin, MELD, PRBC, Early Thrombosis of Hepatic Vessel)

eFigure 2. Kaplan Meier EAF-Free Graft Hazard (A) and Patient Hazard (B) According to the 5 EASE Score Risk Classes

eFigure 3. Trend of C-Statistic During the Post-Operative Period

eFigure 4. Calibration BELT Tests

eMethods. Detailed Description of Statistical Analysis and Workflow to Develop the Final Model

eReferences.

This supplementary material has been provided by the authors to give readers additional information about their work.

© 2020 Avolio AW et al. *JAMA Surg*.

eTable 1. Donor's and Recipient's Data in the Derivation and Validation Sets

	**Derivation set**	**Validation set**			
	Italy (N=1609)	UK (N=538)	*P*-value	*N=2147*	*Missings*
	Median (IQR) or N and (%)	Median (IQR) or N and (%)			*N and %*
**Donor data**					
Donor Age	*65 (51-76)*	*52 (43-61)*	<,001	*2144*	*3 (0,1)*
DCD grafts	*26 (1,6)*	*120 (22,3)*	<,001	*2147*	*0 (0,0)*
Machine Perfusion grafts	*80 (5,0)*	*45 (8,4)*	<,001	*2147*	*0 (0,0)*
**Recipient data**					
Recipient Age	*57 (51-62)*	*56 (49-62)*	*,011*	*2147*	*0 (0,0)*
BMI	*25 (23-28)*	*27 (24-31)*	*<,001*	*2064*	*83 (3,9)*
MELD	*14 (9-20)*	*14 (11-18)*	*,520*	*2147*	*0 (0,0)*
Indications for LT			*<,001*	*2145*	*2 (0,1)*
- *HCV*	*643 (40,0)*	*111 (20,6)*	-	-	-
- *HBV*	*230 (14,3)*	*23 (4,3)*	-	-	-
- *Autoimmune Hepatitis*	*31 (1,9)*	*16 (3,0)*	-	-	-
- *Colestatic Diseases*	*99 (6,2)*	*130 (24,2)*	-	-	-
- *Alcoholic cirrhosis*	*355 (22,1)*	*146 (27,1)*	-	-	-
- *Other indication*	*249 (15,5)*	*112 (20,8)*	-	-	-
HCC co-indication	*715 (44,4)*	*147 (27,3)*	*<,001*	*2146*	*1 (0,0)*
Pre-Transplant Dialysis	*24 (1,5)*	*92 (17,1)*	*<,001*	*2147*	*0 (0,0)*
Pre-Transplant Mechanical Ventilation	*12 (0,7)*	*3 (0,6)*	*,650*	*2147*	*0 (0,0)*
PRBC	*3 (0-6)*	*2 (0-4)*	,114	*2146*	*1 (0,0)*
Vascular thrombosis POD #2-#10 (arterial or portall)	*34 (2,1)*	*58 (10,8)*	<,001	*2147*	*0 (0,0)*
**Donor-recipient match and logistic data**					
D-MELD	825,5 (561,4-1236,6)	691,6 (464,5-975,5)	<,001	*2144*	*3 (0,1)*
Length of hospital stay	15 (10-24)	11 (8-16)	<,001	*2046*	*101 (4,7)*
Main causes of EAF (90 days)	110 (6,8)	41 (7,6)	,505	*2147*	*0 (0,0)*
PDF/PNF/DNF*	32 (29,1)	11 (26,8)			
Vascular Thrombosis	14 (12,7)	7 (17,1)			
Sepsis/MOF**	12 (10,9)	6 (14,6)			
Rejection	13 (11,8)	5 (12,2)			
Other cause	39 (35,5)	12 (29,3)			
LTs at High-volume Center	1144 (71,1)	468 (87,0)	<,001	*2147*	*0 (0,0)*
**Outcome data**					
EAF at 30 days	*63 (3,9)*	*23 (4,3)*	*,713*	*2147*	*0 (0,0)*
EAF at 90 days	*110 (6,8)*	*41 (7,6)*	*,505*	*2147*	*0 (0,0)*
Re-transplant at 90 days	*45 (2,8)*	*27 (5,0)*	*,013*	*2147*	*0 (0,0)*
Death at 90 days	*79 (4,9)*	*30 (5,6)*	*,542*	*2147*	*0 (0,0)*
EASE score	*-3,54±1,55****	*-3,65±1,55****	*,177*	*2140*	*7 (0,3)*

*PDF, Primary dysfunction; PNF, Primary non-function; DNF, Delayed non-function

**MOF, Multiple Organ Failure

***Mean ± SD

© 2020 Avolio AW et al. *JAMA Surg*.

eTable 2. Univariate Analysis and Multivariate Analysis of Factors Predictive of EAF at 90 Days

	UNIVARIATE ANALYSIS at 90 days				MULTIVARIATE ANALYSIS at 90 days			
	**Beta ± SE**	***P*-value**	**OR**	**95% CI**	**Beta ± SE**	***P*-value**	**OR**	**95% CI**
**Donor Age**	-,004 ± 0,006	,525	,996	,986 - 1,007				
**DCD**	,477 ± ,621	,442	1,612	,477 - 5,444				
**Machine perfusion**	,326 ± ,385	,397	1,385	,652 - 2,944				
**Recipient Age**	-0,013 ± ,010	,201	,987	,967 - 1,007				
**MELD**	**,059 ± ,009**	**<,001**	**1,061**	**1,042 - 1,079**	**,044 ± ,014**	**,001**	**1,045**	**1,017 - 1,074**
**HCC T2-T3 (vs no-HCC or HCC-T1)**	**-,339 ± ,193**	***,079***	***,713***	**,488 - 1,040**				
**Dialysis**	**2,272 ± ,340**	**<,001**	**9,701**	**4,979 - 18,901**				
**Mechanical ventilation**	**2,247 ± ,330**	**<,001**	**9,464**	**4,960 - 18,057**				
**PRBC**	**,084 ± ,014**	**<,001**	**1,088**	**1,059 - 1,117**	**,065 ± ,018**	**<,001**	**1,068**	**1,031 - 1,106**
**Intraoperative packing**	**1,844 ± ,379**	**<,001**	**6,321**	**3,007 - 13,290**				
**Re-operation (2-10 day)**	**1,437 ± ,224**	**<,001**	**4,208**	**2,714 - 6,525**				
**Arterial thrombosis (1-10 day)**	**2,077 ± ,459**	**<,001**	**7,979**	**3,243 - 19,634**				
**Portal vein thrombosis (1-10 day)**	**1,425 ± ,673**	**,034**	**4,157**	**1,111 - 15,554**				
**Arterial or venous thrombosis (1-10 day)**	**1,986 ± ,388**	**<,001**	**7,248**	**3,407 - 15,571**	**2,567 ± ,457**	**<,001**	**13,021**	**5,322 - 31,858**
**ln INR**	**1,960 ± ,245**	**<,001**	**7,096**	**4,389 - 11,474**				
**AUC^2 ln AST (1,2,3,7,10 day)**	**,001 ± ,000**	**<,001**	**1,001**	**1,001 - 1,001**	**,000534 ± ,000*157***	**,001**	**1,001**	**1,000 - 1,001**
**AUC ln platelets (1,3,7,10 day)**	**-,201 ± ,020**	**<,001**	**,818**	**,786 - ,850**	**-,093 ± ,026**	**<,001**	**,911**	**,867 - ,958**
**AUC ln bilirubin (1,3,7,10 day)**	**,019 ± ,002**	**<,001**	**1,019**	**1,015 - 1,023**				
**SLOPE ln AST (1,2, 3,7,10 day)**	-,430 ± ,531	,418	,650	,230 - 1,841				
**SLOPE ln platelets (1,3,7,10 day)**	**11,863 ± 1,188**	**<,001**	**,000**	**,000 - ,000**	**-7,766 ± 1,388**	**<,001**	**,000**	**,000 - ,006**
**SLOPE ln bilirubin (1,3,7,10 day)**	**1,206 ± ,151**	**<,001**	**3,339**	**2,482 - 4,492**	**,795 ± ,155**	**<,001**	**2,214**	**1,635 - 2,999**
**AUC ln ALT (1,2,3,7,10 day)**	**,086 ± ,015**	**<,001**	**1,089**	**1,058 - 1,121**				
**AUC^2 ln ALT (1,2,3,7,10 day)**	**,001 ± ,000**	**<,001**	**1,001**	**1,001 - 1,001**				
**SLOPE ln ALT (1,2,3,7,10 day)**	**-2,060 ± ,745**	**,006**	**,127**	**,030 - ,549**				
**Large volume Center (vs medium)**	**-,491 ± ,191**	**,010**	**,612**	**,421 - ,889**	**-,402 ± ,254**	**,114**	**,669**	**,406 - 1,102**
**2017 year**	,180 ± ,186	,331	1,198	,832 - 1,723				

Multivariate analysis achieved a Hosmer Lemeshow goodness of fit equal to 0,883; beta of constant equal to -0,958±1,080; OR of constant equal to 0,384
Significant values are in bold. Values not significant but with a P value <.2 are in italic characters.

© 2020 Avolio AW et al. *JAMA Surg*.

eTable 3. C-Statistics of EASE Score (Final Model 9) and Other Models (5, 6, 7, 8) at 90 Days in the Derivation Set and in the External Validation Set

	derivation set		external validation set	
	**C-stat**	**95% CI**	**C-stat**	**95% CI**
MODEL 9: EASE-score	0,868	0,829-0,908	0,778	0,689-0,867
model 5: the thrombosis covariate was not included in the logistic model	0,854	0,813-0,895	0,709	0,612-0,806
model 6: the thrombosis covariate was not included; DCD-grafts MP-grafts were excluded	0,840	0,794-0,886	0,722	0,626-0,817
model 7: grafts with thrombosis were excluded	0,852	0,811-0,893	0,704	0,606-0,802
model 8: grafts with thrombosis, DCD-grafts and MP-grafts were excluded	0,855	0,815-0,895	0,707	0,610-0,804

© 2020 Avolio AW et al. *JAMA Surg*.

eTable 4. Representative Cases With Relative EASE Scores and Allograft Outcomes

	Pt #520	Pt #1721	Pt #598	Pt #877	Pt #1735
**MELD** (+0.044)	25	14	**40**	19	**30**
**PRBC** (+0.065)	5	4	**11**	**12**	4
**Thrombosis** (+2.567)	**yes (arterial)**	**yes (portal)**	no	no	no
**AST day 1**	253	613	**11139**	583	954
**AST day 2**	199	740	**3438**	517	751
**AST day 3**	130	125	**1189**	396	674
**AST day 7**	34	212	**90**	50	89
**AST day 10**	14	40	**79**	74	56
**PLT day 1**	32	29	20	60	**20**
**PLT day 3**	41	63	67	47	**27**
**PLT day 7**	54	63	33	49	**12**
**PLT day 10**	136	103	91	92	**3**
**Bilirubin day 1**	8.8	5.1	6.9	**7.9**	**15.4**
**Bilirubin day 3**	4.7	5.8	4.5	**10.0**	**16.5**
**Bilirubin day 7**	4.4	6.9	11.2	**22**	**25.3**
**Bilirubin day 10**	3	9.3	11.2	**35**	**37.0**
**High volume Center** (-0.402)	**no**	yes	**no**	**no**	yes
AUC^2 AST (+0.000534)	1334	2559	2789	1983	2312
AUC PLT (-0.093)	35.9	35.2	34.6	36.0	23.2
Slope PLT (-7.766)	0.15	0.12	0.11	0.05	-0.22
Slope Bilirubin (+0.735)	-0.534	0.442	0.684	3.055	2.409
EASE score	***-1.034***	***-0.444***	***0,596***	***0.356***	***3.186***
EASE class	*3*	*4*	*5*	*5*	*5*
EASE risk of failure	***26.2±1.5%***	***39.1±2.9%***	***64.5±3.6%***	***69.6±4.8%***	***96.0±5.9%***
Failure (day of failure)	*no*	*no*	*yes (28 POD)*	*yes (46 POD)**	*yes (76 POD)*
Death (day of death)	*no*	*no*	*Yes (28 POD)*	*yes (68 POD)**	*Yes (76 POD)*

Seventeen data-entries and related 8 risk factors (beta-coefficients between brackets) are displayed. The EASE score reliably predicts EAF also in cases whose outcome is unexpected according to the clinical course and/or previous scores.

*This patient died after being re-transplanted.

© 2020 Avolio AW et al. *JAMA Surg*.

eTable 5. C-statistics of EASE Score (Which Is Calculated at 90 Days) and Other Prognostic Scores in the Derivation Set, EASE Score Shows the Highest C-Statistic at 90 Days

	EASE Derivation set (90 d)				EASE Internal Validation set (90 d)				EASE Derivation set (30 d)	
	C-stat	95% CI	Chi2	p-value	C-stat	bias	bias SE	95% CI.	C-stat	95% CI
EASE-score (ref)	0,868	0,826-0,911			0,868	-0,0008	0,021	0,827-0,910	0,927	0,887-0,967
MEAF[17]	0,727	0,667-0,788	22,848	<,001	0,727	-0,0018	0,029	0,670-0,790.	0,829	0,771-0,886
L-GrAFT[20]	0,714	0,647-0,782	27,031	<,001	0,714	0,7142	-0,002	0,639-0,779.	0,798	0,717-0,879
EAD[16]	0,699	0,632-0,753	62,236	<,001	0,472	-0,0015	0,041	0,391-0,562	0,770	0,702-0,839
D-MELD[29]	0,602	0,537-0,667	53,614	<,001	0,602	0,0001	0,034	0,536-0,668	0,594	0,510-0,678
New ET-DRI[30]	0,552	0,488-0,616	76,550	<,001	0,551	0,0010	0,033	0,486-0,616	0,527	0,437-0,617
DRI[6]	0,529	0,464-0,592	91,974	<,001	0,528	-0,0019	0,032	0,466-0,590	0,530	0,444-0,615

EASE score shows the highest C-statistic at 90 days.
The *P* values refer to the comparison of the indicated score against EASE.
As shown by absence of overlap of 95% CI between EASE score and other scores, EASE has a high discrimination ability.

© 2020 Avolio AW et al. *JAMA Surg.*

eFigure 1. Changes in Cox-Estimated Hazard Ratio (HR) of Significant Covariates (AUC^2 in PLT, Slope in PLT, Slope in Bilirubin, MELD, PRBC, Early Thrombosis of Hepatic Vessel). MELD was significant at POD 2-30, 2-60, and 2-90 evaluation times. The red-dashed line represents the significance level.

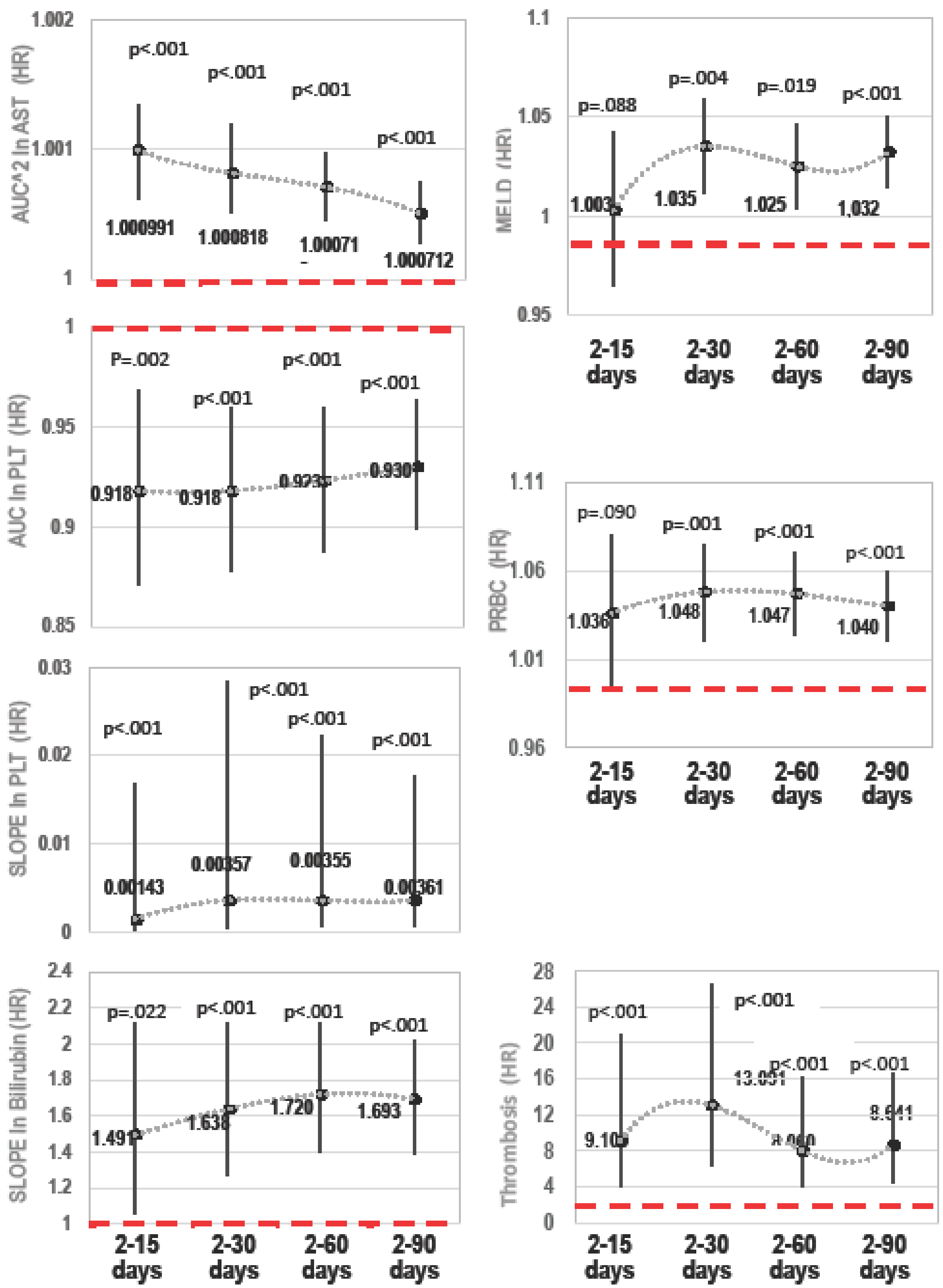

© 2020 Avolio AW et al. *JAMA Surg*.

eFigure 2. Kaplan Meier EAF-free graft hazard (A) and patient hazard (B) according to the 5 EASE score risk classes. The dashed line indicates the highest hazard of extremely high-risk class patients. Numbers at risk and ranges of classes are reported below.

A: Graft Hazard

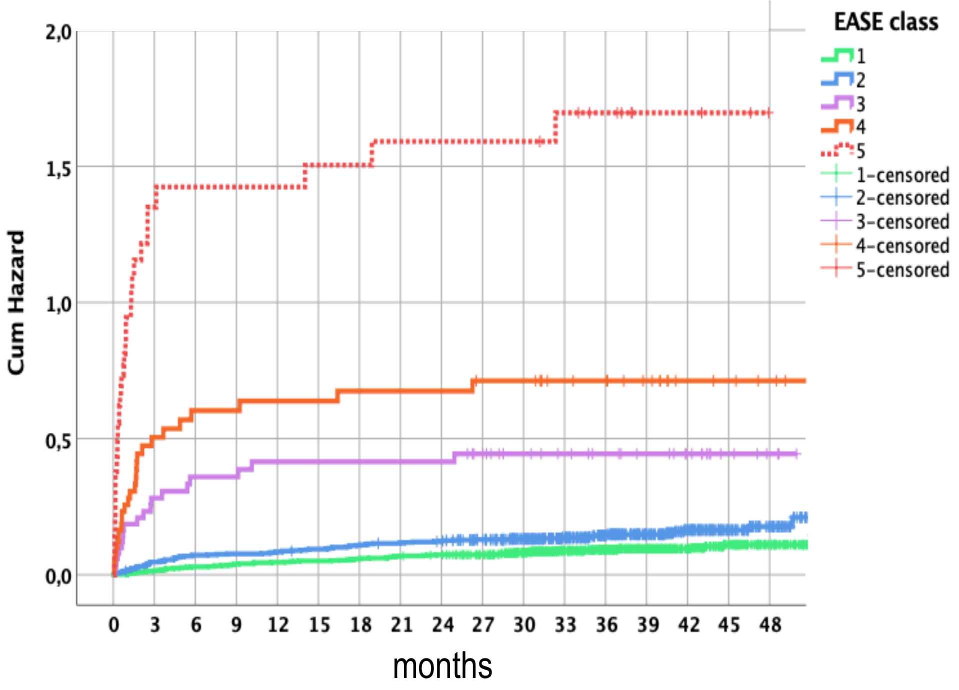

	0	3	6	12	18	24	30	36	42	48
class 1	810	799	787	774	765	751	667	448	245	84
class 2	639	609	594	587	572	561	486	326	166	61
class 3	53	40	37	35	35	35	26	20	13	3
class 4	53	32	29	28	27	27	25	19	8	5
class 5	54	14	13	13	12	11	11	7	3	1
	1609	1494	1460	1437	1411	1385	1215	820	435	154

B: Patient Hazard

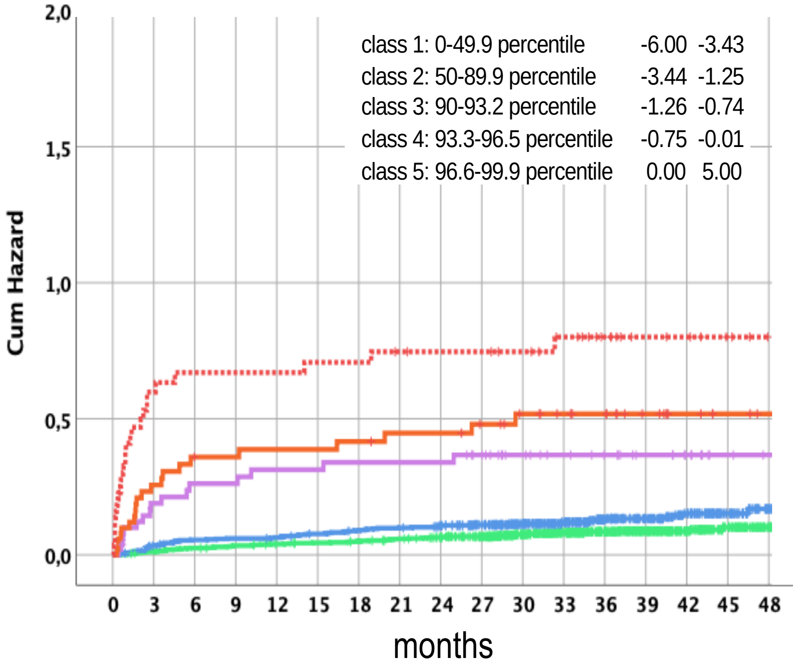

	0	3	6	12	18	24	30	36	42	48
class 1	810	781	790	778	768	756	671	452	248	87
class 2	639	617	604	599	580	569	493	331	170	63
class 3	53	44	41	39	38	38	28	21	13	3
class 4	53	41	37	36	35	34	30	23	9	5
class 5	54	30	27	27	26	24	22	14	6	1
	1609	1513	1499	1479	1447	1421	1244	841	446	159

© 2020 Avolio AW et al. *JAMA Surg*.

eFigure 3. Trend of C-Statistic During the Post-Operative Period. The four evaluations (day 15, day 30, day 60, day 90) show the persistence of excellent C-statistic although the reasonable decrease from day 15 to day 90.

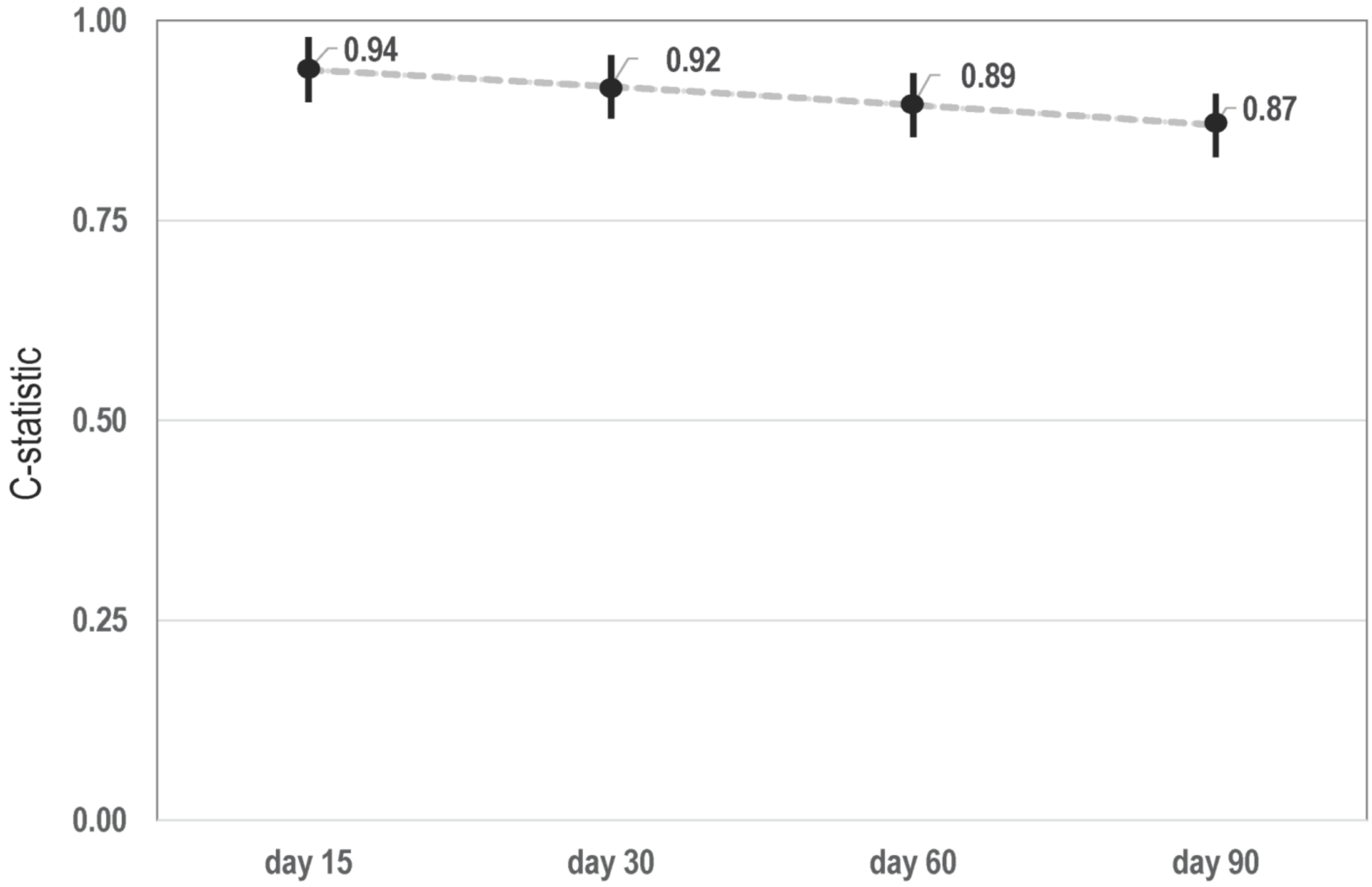

© 2020 Avolio AW et al. *JAMA Surg*.

eFigure 4. Calibration BELT Tests.

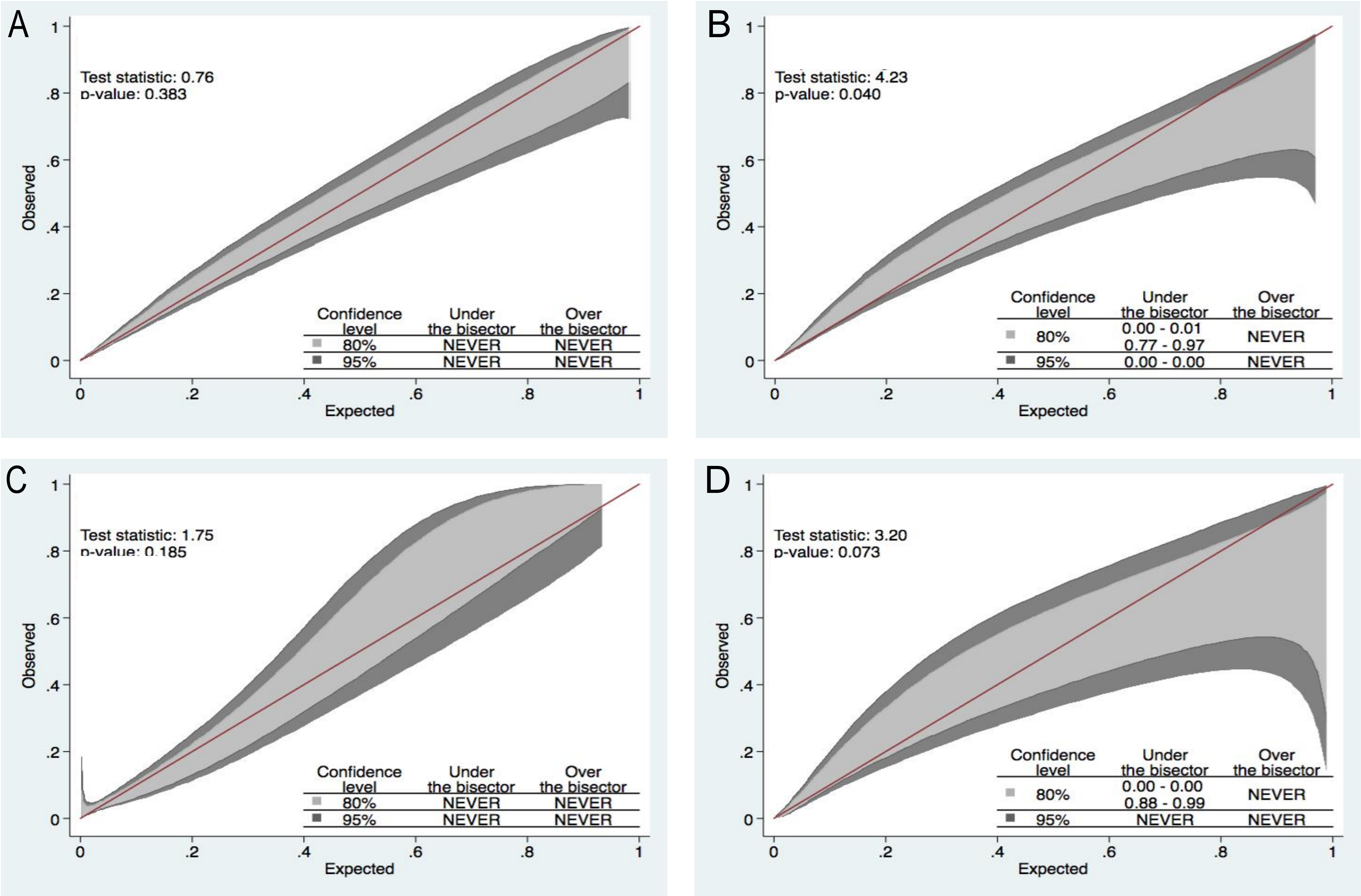

Panels A and B refer to EASE score tested at 90 and 30 days in the derivation set. Panel C and D refer to EASE score tested at 90 and 30 days in the external validation set. The predictions of the model do not deviate from the observed rate in the derivation sample (that is, that the model's internal calibration is acceptable). However, although the C-statistics demonstrated a good discrimination ability of the EASE score at 90-days and 30-days, the output of calibration BELT test suggested that the fitted model at 30 day was not well calibrated (i.e. p values lower than 0.1, belts under the bisector for high predicted probabilities).

© 2020 Avolio AW et al. *JAMA Surg*.

eMethods. Detailed Description of Statistical Analysis and Workflow to Develop the Final Model

1.Statistical analysis

The study was performed according to current recommendations for retrospective observational analysis reporting in transplant population.[1-2] Continuous variables were presented as medians and interquartile ranges (IQR) or means ± standard deviations (SD), whilst categorical variables were summarized as numbers and percentages. Categories of patients who could be confounders due to peculiarities and/or low prevalence were excluded (Figure 1). Missing data were not managed by imputation methods because of their exiguous number (eTable 1).

Graft and patient survival curves were performed according to Kaplan-Meier and compared using the log- rank test. The goodness of fit was assessed using the Hosmer-Lemeshow test.[3] We evaluated also the calibration of the final model using the Calibration-BELT test of the final model.[4] In the derivation set, the C-statistic comparison of the final model at 90 days with Model for Early Allograft Failure (MEAF),[5] L-GrAFT,[6] EAD,[7] Donor age x Model for End-stage Liver Disease (D-MELD),[8] new Theoretical Euro-Transplant Donor Risk Index (ET-DRI),[9] and Donor Risk Index (DRI)[10] was performed through non-parametric method.[11]

The *P* value <.05 was considered significant. Statistics were performed using SPSS (ver. 25.0) and STATA (ver. 14.0) packages.

2. Work-flow to develop the final model

We initially replicated the methodology adopted in the seminal L-GrAFT study.[6] This score was derived through a kinetic approach using the area under the curve (AUC), direction and steepness of the curve (SLOPE). AUC and SLOPE were calculated using 10 evaluations (one a day from day 1 to 10). This methodology was adopted for AST, platelets and bilirubin. The highest value of INR, recorded from day 1 to 10, was included. The logarithmic trapezoidal method was used to calculate AUC and SLOPE for AST and platelets, while standard linear trapezoidal method was used for bilirubin.[6]

In this study, we aimed to build a comprehensive model available at the 10th postoperative day. An extensive set of variables was considered, including pre-operative and intraoperative parameters. Due to the time-dependence incidence of EAF, variables were first analyzed by univariate Cox regression, adopting the same methodology used to develop the L-GrAFT. In addition to other significant parameters, not relevant for subsequent analysis, PRBC, THV, AST-AUC^2, platelets-AUC, platelets-SLOPE, and bilirubin-SLOPE were significant at all time spans. MELD was significant at POD 2-30, 2-60, and 2-90 evaluation times (eFigure 1).

Single values of AUC and SLOPE were calculated for each case. Some variables in the original L- GrAFT model were expressed as their squared forms, and for these we adopted the square elevation. Such variables were then analyzed by univariate and multivariate logistic regression. Only variables with a P value <.2 at univariate logistic regression were included in multivariate analysis. Interestingly, not all the variables included in the L-GrAFT were significant.

We initially tested the same beta-coefficients of the original L-GrAFT model derived from 40 data entries validating L-GrAFT in our population. Following evaluation of the entire set of lab data we reduced the number of entries by recording only data at specified PODs.

In details, the number of lab data entries was reduced (fixed POD determinations instead of each day determinations from POD 1 to POD 10). In total, there were 4 entries for bilirubin and 4 entries for PLT (POD 1, 3, 7, and 10) and 5 for AST (POD 1, 2, 3, 7, and 10). The timing of data entries was chosen to best include relevant changes. In order to capture the cytolysis peak, the inclusion of day-2 AST data was necessary. Next, the number of calculated variables was reduced in order to maintain an adequate proportion between parameters and events in the logistic models. Furthermore, additional donor- and recipient-related parameters, not originally included in the L-GrAFT model, were investigated.

In summary, four subsequent logistic models (1, 2, 3, 4) were developed in the derivation set, to reduce the number of data entries, improving C-statistic and including additional factors. Five additional models (5, 6, 7, 8, 9) were tested in order to investigate the impact of THV, DCD and MP grafts in the derivation and validation sets. The models 5-9 were adjusted for Center volume.

© 2020 Avolio AW et al. *JAMA Surg.*

Model 1 (validation of the original L-GrAFT model)
The model (original L-GrAFT model) was based on 40 data entries, namely AST, bilirubin, PLT and INR daily obtained from POD #1 to #10 after LT. The model included the 12 original covariates and the original 12 β-coefficients. The C-statistic obtained by ROC curve analysis was ,74; 95%CI = ,68-,80.

Model 2
This model replicates the Original L-GrAFT (40 data entries resulting in 12 covariates). However, at difference from model 1, the β-coefficients were obtained by logistic regression analysis. The C-statistic obtained by ROC curve analysis was ,84; 95%CI = ,79-,88.

Model 3
This model replicates the original L-GrAFT model (40 data entries resulting in 12 covariates) with the inclusion of 2 additional covariates (MELD+PRBC) for a final number of 42 data entries. All β-coefficients were re-calculated. The C-statistic obtained by ROC curve analysis was ,87; 95%CI = ,83-,91.

Model 4
This model was obtained from AUC and SLOPE parameters obtained from only 13 kinetic entries. In detail, 4 entries for bilirubin and PLT (POD #1, #3, #7, and #10) and to 5 for AST (POD #1, #2, #3, #7, and #10) were included. INR was not included anymore for the absence of significance at logistic analysis. AUC and SLOPE parameters were reduced to 4 covariates (AUC ln AST^2, AUC ln Platelets, SLOPE ln Platelets. SLOPE ln Bilirubin). The C-statistic obtained by ROC curve analysis was ,84; 95%CI = ,79-,82.

All models were internally validated by the bootstrap method. Since we achieved a satisfactory simplification with a reduced number of data entries and similar C-statistic, we started to test the models in the external validation set.

Model 5
This model was derived from model 4 by including MELD and PRBC. The model was adjusted for Center volume. It consisted of 13 kinetic entries (4 entries for bilirubin and PLT, at POD #1, #3, #7, #10 and to 5 for AST at POD #1, #2, #3, #7, #10) which led to 4 covariates + MELD + PRBC + Center volume covariates. The C-statistic obtained by ROC curve analysis was ,85; 95%CI = ,81-,90).

Model 6 (DCD and MP grafts excluded)
This model was similar to Model 5 (13 kinetic entries + MELD + PRBC). THV, DCD and MP grafts were excluded. The model was adjusted for Center volume. It consisted of 13 kinetic entries (4 entries for bilirubin and PLT, at POD #1, #3, #7, #10 and to 5 for AST at POD #1, #2, #3, #7, #10) which led to 4 covariates + MELD + PRBC + Center volume. The C-statistic obtained by ROC curve analysis was ,84; 95%CI = ,79-,89.

Model 7 (THV grafts excluded)
This model was similar to Model 5 (13 kinetic entries + MELD + PRBC). Only THV grafts were excluded. The model was adjusted for Center volume. It consisted of 13 kinetic entries, which led to 4 covariates + MELD + PRBC + Center volume. The C-statistic obtained by ROC curve analysis was ,85; 95%CI = ,81-,89.

Model 8 (THV, DCD, MP grafts excluded)
This model was similar to Model 5 (13 kinetic entries + MELD + PRBC). THV, DCD, and MP grafts were excluded. The model was adjusted for Center volume. It consisted of 13 kinetic entries, which led to 4 covariates + MELD + PRBC + Center volume. The C-statistic obtained by ROC curve analysis was ,85; 95%CI = ,81-,89).

© 2020 Avolio AW et al. *JAMA Surg*.

Model 9 (THV, DCD, MP grafts included)

The model was obtained from model 4 by including additional covariates. Odd ratios and confidence intervals are detailed in eTable 2. THV, DCD, and MP grafts were included. The model included 13 kinetics entries (4 entries for bilirubin and PLT, at POD #1, #3, #7, #10 and to 5 for AST at POD #1, #2, #3, #7, #10), MELD, PRBC and THV. The total number of variables is 7 (bilirubin, PLT, AST, MELD, PRBC, THV, Center volume), however the number of covariates included is 8 because PLT is entered as AUC and as SLOPE. The model was adjusted for Center volume. The C-statistic obtained by ROC curve analysis was ,87; 95%CI=,83-,91.

The final simplified comprehensive model (model 9) was selected based on the low number of data entries (N=17) and the highest C-statistics in both derivation and validation sets. The score was named EASE (Early Allograft Simplified Estimation) and included all graft categories, encompassing all possible scenarios for EAF prediction. The AUC curves of the EASE score and all other models were reported in Figure 2.

© 2020 Avolio AW et al. *JAMA Surg*.

eReferences

1. von Elm E, Altman DG, Egger M, Pocock SJ, Gøtzsche PC, Vandenbroucke JP. The Strengthening the Reporting of Observational Studies in Epidemiology (STROBE) statement: guidelines for reporting observational studies. Int J Surg 2014;12:1495-1499.
2. Kalil AC, Mattei J, Florescu DF, Sun J, Kalil RS. Recommendations for the assessment and reporting of multivariable logistic regression in transplantation literature. Am J Transplant 2010;10:1686-1694.
3. Hosmer DW, Hosmer T, Le Cessie S, Lemeshow S, Finazzi S, Bertolini G. A comparison of goodness-of-fit tests for the logistic regression model. Stat Med 1997;16:965-980.
4. Nattino G, Lemeshow S, Philllips G Assessing the calibration of dichotomous outcome models with the calibration belt The Stata Journal 2017; 17: 1003-1014
5. Pareja E, Cortes M, Hervás D, Mir J, Valdivieso A, Castell JV, et al. A score model for the continuous grading of early allograft dysfunction severity. Liver Transpl 2015;21:38-46.
6. Agopian VG, Harlander-Locke MP, Markovic D, Dumronggittigule W, Xia V, Kaldas FM et al. Evaluation of Early allograft Function Using the Liver Graft Assessment Following Transplantation Risk Score Model. JAMA Surg 2018; 153:436-444.
7. Olthoff KM, Kulik L, Samstein B, Kaminski M, Abecassis M, Emond J, et al. Validation of a current definition of early allograft dysfunction in liver transplant recipients and analysis of risk factors. Liver Transpl 2010;16:943-949.
8. Avolio AW, Cillo U, Salizzoni M, De Carlis L, Colledan M, Gerunda GE, et al. Balancing donor and recipient risk factors in liver transplantation: the value of D-MELD with particular reference to HCV recipients. Am J Transplant 2011;11:2724-2736.
9. Braat AE, Blok JJ, Putter H, Adam R, Burroughs AK, Rahmel AO, et al. The Eurotransplant donor risk index in liver transplantation: ET-DRI. Am J Transplant 2012;12:2789-2796.
10. Feng S, Goodrich NP, Bragg-Gresham JL, Dykstra DM, Punch JD, DebRoy MA, et al. Characteristics associated with liver graft failure: the concept of a donor risk index. Am J Transplant 2006;6:783-790.
11. DeLong ER, DeLong DM, Clarke-Pearson DL. Comparing the areas under two or more correlated receiver operating characteristic curves: a non parametric approach. Biometrics 1988;44:837-845.

© 2020 Avolio AW et al. *JAMA Surg*.
